# Comparison of blue laser imaging and light‐emitting diode‐blue light imaging for the characterization of colorectal polyps using the Japan narrow‐band imaging expert team classification: The LASEREO and ELUXEO COLonoscopic study

**DOI:** 10.1002/deo2.245

**Published:** 2023-05-18

**Authors:** Masahiro Okada, Naohisa Yoshida, Hiroshi Kashida, Yoshikazu Hayashi, Satoshi Shinozaki, Shiori Yoshimoto, Toshihiro Fujinuma, Hirotsugu Sakamoto, Keijiro Sunada, Yuri Tomita, Osamu Dohi, Ken Inoue, Ryohei Hirose, Yoshito Itoh, Yoriaki Komeda, Ikue Sekai, Natsuki Okai, Alan Kawarai Lefor, Hironori Yamamoto

**Affiliations:** ^1^ Department of Medicine, Division of Gastroenterology Jichi Medical University Tochigi Japan; ^2^ Department of Molecular Gastroenterology and Hepatology Kyoto Prefectural University of Medicine, Graduate School of Medical Science Kyoto Japan; ^3^ Department of Gastroenterology and Hepatology Kindai University Osaka Japan; ^4^ Shinozaki Medical Clinic Tochigi Japan

**Keywords:** blue light imaging, Japan narrow band imaging expert team, laser endoscopy, light‐emitting diode, narrow‐band light

## Abstract

**Objectives:**

Although the laser light is optically ideal for producing narrow‐band light, it has not been used in some areas of the world. Endoscopic light sources using light‐emitting diodes (LEDs) are used worldwide. The purpose of this study was to compare blue laser imaging (laser‐BLI) and LED‐blue light imaging (LED‐BLI) for the characterization of colorectal polyps using the Japan narrow band imaging expert team (JNET) classification.

**Methods:**

Colorectal lesions were prospectively examined using magnifying narrow‐band light generated by a laser (laser‐BLI) or LEDs (LED‐BLI). Twelve endoscopists (six non‐experts and six experts from three institutions) evaluated each still‐magnified image of lesions using the JNET classification.

**Results:**

Seven hundred and fifty‐six images from 63 lesions were reviewed. The mean polyp size was 24.5 ± 13.4 mm. Histopathology included 13 serrated lesions and 50 neoplasms. The rate of agreement between laser‐BLI and LED‐BLI using the JNET classification was 92.5% (699/756). The weighted κ‐statistic was 0.99. The percentages of “almost similar” comparing scores of surface patterns, vessel patterns, and brightness among all endoscopists were 95.4%, 95.9%, and 95.0%, respectively.

**Conclusions:**

This multicenter study demonstrates that the rate of agreement between laser‐BLI and LED‐BLI using the JNET Classification is very high. The surface patterns, vessel patterns, and brightness are almost similar.

## INTRODUCTION

Narrow‐band light is a revolutionary endoscopic image enhancement technology for characterizing gastrointestinal lesions. The first narrow‐band light endoscope unit was launched as the narrow‐band imaging (NBI) endoscopy system by Olympus Corp. (Tokyo, Japan) in 2005.^1^ When performing magnifying NBI endoscopy, most gastrointestinal tumors can be characterized without using chemical materials such as indigo carmine, iodine, acetic acid, or crystal violet. Magnifying NBI colonoscopy has been accepted as a standard endoscopic examination for colorectal lesions in Japan.

Fujifilm Corp. (Tokyo, Japan) developed an original narrow‐band light system using a laser light source and launched it as blue laser imaging (BLI) in 2012. The laser light source generates two wavelengths of light at 410 ± 10 and 450 ± 10 nm.^2^ Laser light at a wavelength of 410 nm is absorbed by hemoglobin in the micro‐vessels on the surface of polyps and enhances the vessels. Laser light at a wavelength of 450 nm excites phosphors to produce white light.^3^ Although magnifying laser BLI had as good diagnostic ability as magnifying NBI,^2^ there were some places such as the United States and European countries where the use of laser light for endoscopy was not approved. Therefore, Fujifilm Corp. developed a new narrow‐band light system using light‐emitting diodes (LEDs) as an endoscopic light source and called it Blue “Light” Imaging in 2017. To avoid possible confusion, we defined BLI using laser light as laser‐BLI and BLI using LEDs as LED‐BLI. The LED light source emits four wavelengths of light that are blue‐violet, blue, green, and red. The blue‐violet LED has a wavelength of around 410 nm and is dedicated to NBI.^3^, ^4^

It is still unclear if magnifying LED‐BLI has as good characterization capability as magnifying laser‐BLI. In the present study, we compared the image quality for determining the Japan NBI expert team (JNET) classification.^5^ of magnifying laser‐BLI and magnifying LED‐BLI as a sub‐group analysis of the LASEREO and ELUXEO COLonoscopic comparison study (LECOL study) which showed non‐inferiority of linked‐color imaging and white‐light imaging comparing LED and laser colonoscopy.^6^

## METHODS

This is a sub‐group analysis of the LECOL study, which was reported previously.^6^ Lesions were prospectively observed with BLI using laser and LED colonoscopy from January 2021 to August 2021 in two institutions (Kyoto Prefectural University Hospital and Jichi Medical University Hospital). Consecutive cases treated with endoscopic submucosal dissection (ESD) were enrolled and these patients underwent endoscopy twice including precheck endoscopy and therapeutic endoscopy. Other small lesions close to the main lesion were also observed. Patients with colorectal lesions that had been examined at the first precheck laser or LED colonoscopy observation before ESD were enrolled. These lesions were observed again with a second laser or LED colonoscopy, using the modality which was not used in the precheck colonoscopy, on the day of ESD. Moderately magnified BLI pictures that represent the character of each lesion were taken using laser or LED colonoscopy. The pictures were taken of each lesion under as similar conditions as possible concerning the angle and distance of the lesion and the amount of insufflation using the tablet‐image comparison method as described in a previous report.^7^ Pictures obtained with the first procedure were saved digitally. Finally, a pair of magnifying pictures using both laser‐BLI and LED‐BLI, very close to each other, were prepared for each lesion. The interval between precheck day and ESD day was within 1 month.

Eighty‐three lesions observed with both laser and LED colonoscopy were prospectively collected during the LECOL study period. Two endoscopists (Naohisa Yoshida and Yuri Tomita) inspected the quality of each picture and excluded 20 lesions due to a lack of perfect resemblance to the paired pictures. Finally, 63 laser‐BLI pictures and 63 LED‐BLI pictures were independently evaluated by a total of 12 endoscopists from three institutions including Jichi Medical University, Kyoto Prefectural University of Medicine, and Kindai University. Neither white‐light pictures nor non‐magnifying pictures were included. This study was conducted in accordance with the World Medical Association Helsinki Declaration. This study was also approved by the Institutional Review Board of Jichi Medical University (No. 20–105) and Kyoto Prefectural University of Medicine (ERB‐C‐1824).

### Laser endoscopes and LED

The laser endoscopic system (LASEREO; Fujifilm) consisting of an LL‐7000 light source, VP‐7000 processor, and EC‐L600ZP, EC‐L600ZP7, EC‐L600ZP7/L (Fujifilm) colonoscopes and the LED endoscopic system (ELUXEO; Fujifilm) consisting of the BL‐7000 light source, VP‐7000 processor, and an EC‐760ZP‐V/M colonoscope were used in this study. Structural enhancement for the laser‐BLI and the LED‐BLI was set to level B8. Color enhancement was set to level C2 (Table 1).

**TABLE 1:** Specifications of each system.

					Enhancement	
	System	Light source	Processor	Endoscope	Structure	Color
Laser‐BLI	LASEREO	LL‐7000	VP‐7000	EC‐L600ZP EC‐L600ZP7 EC‐L600ZP7/L	B8	C2
LED‐BLI	ELUXEO	BL‐7000	VP‐7000	EC‐760ZP‐V/M	B8	C2

Abbreviations: BLI, blue light imaging; Laser‐BLI, blue laser imaging; LED, light‐emitting diode.

### Endoscopic pictures

**FIGURE 1:**
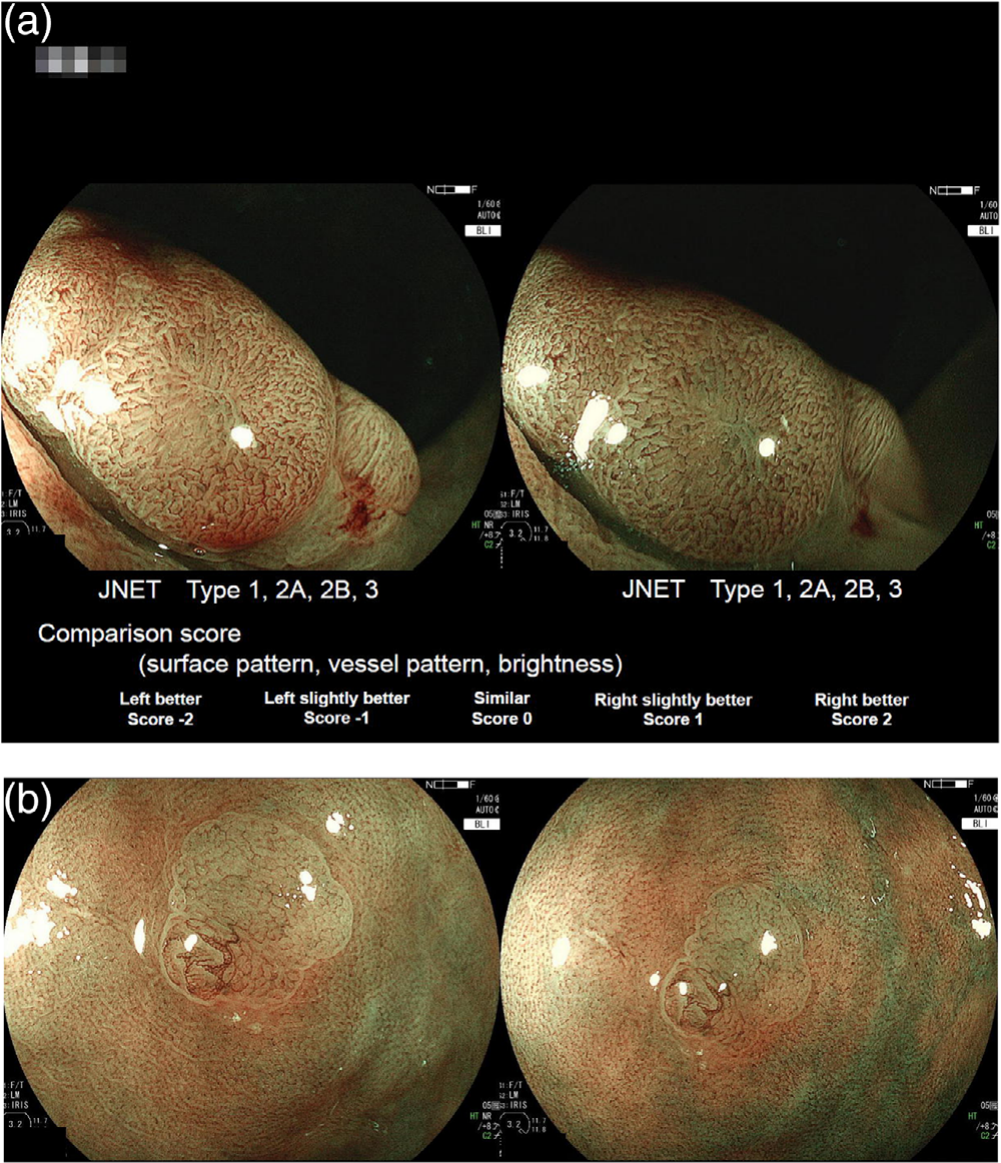
Sample paired pictures obtained blue laser imaging (laser‐BLI) and using light‐emitting diode‐blue light imaging (LED‐BLI). (a) A 22 mm low‐grade adenoma in the rectum. Evaluators assigned one of the four Japan narrow band imaging expert team (JNET) classifications for each image and assigned one of the five comparison scores for characteristics including surface pattern, vessel pattern, and brightness. (b) A 4 mm sessile serrated lesion with dysplasia in the rectum.

Each of the 126 pictures of the 63 lesions taken with LED‐BLI and laser‐BLI was evaluated by the 12 endoscopists. Each pair of pictures was randomly arranged side by side (Figure 1). Whether the picture was taken with laser or LED was blinded to the evaluators. The endoscopists classified each of the paired pictures of each lesion into JNET classifications of Type 1, Type 2A, Type 2B, or Type 3 with a confidence level (high or low). The evaluators also scored the surface pattern, vessel pattern, and brightness of each pair of the BLI pictures as “LED better”, “LED slightly better”, “similar”, “laser slightly better”, and “laser better”.

### Evaluators

Each of the three participating institutions provided two experts who had performed over 5,000 colonoscopies and over 50 magnifying colonoscopies and non‐experts who had performed fewer colonoscopies. The twelve endoscopists had not seen any of the pictures of lesions in this study before the evaluation.

### Study outcomes

The primary outcome of this study was an agreement with the JNET classification obtained using laser‐BLI and LED‐BLI.

### Histopathological diagnosis

Polyp size was defined by its maximum diameter and was estimated from the resected‐specimen size, compared with snares and biopsy forceps. Polyps were classified into polypoid and non‐polypoid types, according to the Paris classification. The endoscopically resected specimens of the lesions were fixed in 10% formalin and evaluated pathologically. Histopathological diagnosis was performed by clinical pathologists employed at each facility according to the classification by the World Health Organization; serrated lesion, low‐grade adenoma, high‐grade dysplasia, and T1 cancer. Serrated lesions were classified into sessile serrated lesion, sessile serrated lesion with dysplasia, traditional serrated adenoma, and hyperplastic polyp.

### Statistical analyses

The weighted kappa value was calculated to assess the agreement of the JNET classification between laser‐BLI and LED‐BLI. When the kappa value is less than 0.4, 0.4–0.6, 0.6–0.8, and over 0.8, their agreements are rated as poor, moderate, good, and excellent, respectively. The Chi‐square test or Fisher's exact test was used to compare categorical variables. A *p*‐value <0.05 was considered statistically significant. EZR (Saitama Medical Center, Jichi Medical University, Saitama, Japan) was used for all statistical analyses.^8^

## RESULTS

The characteristics of 63 lesions from 55 patients in the present study were shown in Table 2. The mean lesion size was 24.5 ± 13.4 mm. The number of right‐sided (from cecum to transverse colon) and left‐sided (from descending colon to rectum) lesions were 30 (48%) and 33 (52%), respectively. All 63 lesions were non‐polypoid.

**TABLE 2:** Lesion characteristics.

Characteristics	Data
Number of patients, *n*	55
Age, mean ± SD, years	67.9 ± 10.8
Male, *n*	26 (47%)
Number of lesions, *n*	63
Tumor size, mean ± SD	24.5 ± 13.4
Right‐sided/left‐sided colon, *n*	30 (48%)/33 (52%)
Polypoid/non‐polypoid, *n*	0 (0%)/63 (100%)
Histopathology	
Hyperplastic polyp	5 (8%)
Sessile serrated lesion	6 (10%)
Sessile serrated lesion with dysplasia	2 (3%)
Low‐grade adenoma	26 (41%)
High‐grade dysplasia	11 (17%)
T1a	6 (10%)
T1b	7 (11%)

Abbreviations: SD, standard deviation; T1a, slightly invasivesubamucosal cancer (<1000 μm); T1b, deeply invasive submucosal cancer (1000 μm).

The rate of agreement of the JNET classification using images obtained using laser‐BLI and LED‐BLI for all lesions was 92.5% (699/756). The weighted kappa value was 0.99, indicating excellent agreement (Table 3). Regarding the endoscopists’ experience, weighted kappa values of the JNET classifications between laser‐BLI images and LED‐BLI were 1.00 and 0.98 among experts and non‐experts, respectively (Table 3). For lesions scored with “high confidence,” the weighted kappa values of the JNET classification were 1.00, 1.00, and 1.00 for the total, experts, and non‐experts, respectively (Table 4). All weighed kappa values indicate excellent agreement. Regarding if the lesions were neoplasms or not, the weighted kappa values of the JNET classification between laser‐BLI and LED‐BLI images were 0.99 and 0.99 for non‐neoplasms and neoplasms, respectively (Table 5).

**TABLE 3:** Agreement of Japan narrow band imaging expert team classifications using blue laser imaging and light‐emitting diode‐blue light imaging images.

		Laser			
Total/experts/non‐experts		Type 1	Type 2A	Type 2B	Type 3
LED	Type 1	138/76/62	2/1/1	1/0/1	0/0/0
	Type 2A	5/0/5	234/113/121	21/5/16	1/0/1
	Type 2B	1/0/1	10/4/6	266/136/130	7/2/5
	Type 3	1/0/1	0/0/0	8/4/4	61/37/24

*Note*: Weighted kappa value (total/experts/non‐experts) = 0.99/1.00/0.98.

Abbreviation: LED, light‐emitting diode.

**TABLE 4:** Agreement of Japan narrow band imaging expert team classifications using blue laser imaging and light‐emitting diode‐blue light imaging images scored with “high‐confidence.”

		Laser			
Total/experts/non‐experts		Type 1	Type 2A	Type 2B	Type 3
LED	Type 1	100/62/38	0/0/0	0/0/0	0/0/0
	Type 2A	1/0/1	161/90/71	4/2/2	0/0/0
	Type 2B	0/0/0	0/0/0	152/80/72	1/1/0
	Type 3	0/0/0	0/0/0	2/1/1	44/29/15

*Note*: Weighted kappa value (total/experts/non‐experts) = 1.00/1.00/1.00.

Abbreviation: LED, light‐emitting diode.

**TABLE 5:** Agreement of Japan narrow band imaging expert teamclassifications using blue laser imaging and light‐emitting diode‐blue light imaging images for non‐neoplasms and neoplasms.

		Laser			
Non‐neoplasms/neoplasms		Type 1	Type 2A	Type 2B	Type 3
LED	Type 1	99/39	2/0	0/1	0/0
	Type 2A	2/3	21/213	2/19	0/1
	Type 2B	1/0	2/8	1/265	0/7
	Type 3	0/1	0/0	1/7	1/60

*Notes*: Weighted kappa value (non‐neoplasms/neoplasms) = 0.99/0.99; Non‐neoplasms: hyperplastic polyp and sessile serrated lesion; Neoplasms: sessile serrated lesion with dysplasia and adenoma, and cancer.

Abbreviation: LED, light‐emitting diode.

**FIGURE 2:**
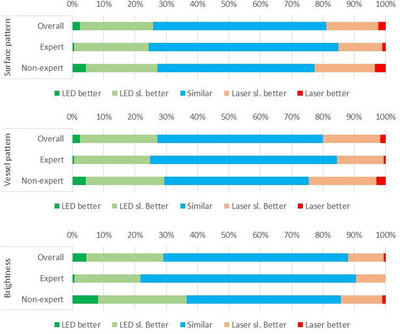
Comparison scores of each characteristic.

**FIGURE 3:**
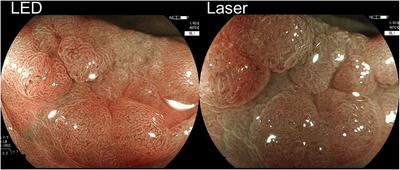
Representative paired pictures assigned to the “better” category, to whether using blue laser imaging (laser‐BLI) or light‐emitting diode‐blue light imaging (LED‐BLI). A 20 mm low‐grade adenoma in the transverse colon. The LED‐BLI picture was evaluated as having better brightness than the laser‐BLI picture.

The comparison scores for surface patterns, vessel patterns, and brightness obtained from images using laser‐BLI and LED‐BLI were shown in Table 6 and Figure 2. If “LED slightly better”, “similar”, and “laser slightly better” are combined into one group referred to as “almost similar”, the percentages of the combined “almost similar” group for surface patterns, vessel patterns, and brightness were 95.4%, 95.9%, and 95.0%, respectively. The percentages among experts were 98.4%, 98.9%, and 99.2%, respectively. The percentages among non‐experts were 92.3%, 92.9%, and 90.7%, respectively. Experts judged as “almost similar” significantly more frequently than non‐experts for the surface pattern (*p* = 0.00), vessel pattern (*p* = 0.00), and brightness (*p* = 0.00). When discussing which light source is better, LED‐BLI was "better" than laser‐BLI for brightness as judged by non‐experts (8.2% [31/378] vs. 1.1% [4/378]). Seventeen of 31 assignments of "better" in LED‐BLI were adenomas (Figure 3).

**TABLE 6:** Comparison scores.

		LED better	LED sl. better	Similar	Laser sl. better	Laser better
Surface pattern	Overall	18	177	418	126	17
			95.4% (721/756)			
	Expert	2	90	229	53	4
			98.4% (372/378)			
	Non‐expert	16	87	189	73	13
			92.3% (349/378)			
Vessel pattern	Overall	18	187	399	139	13
			95.9% (725/756)			
	Expert	2	92	225	57	2
			98.9% (374/378)			
	Non‐expert	16	95	174	82	11
			92.9% (351/378)			
Brightness	Overall	34	186	446	86	4
			95.0% (718/756)			
	Expert	3	79	260	36	0
			99.2% (375/378)			
	Non‐expert	31	107	186	50	4
			90.7% (343/378)			

*Notes*: Right‐sided, cecum to the transverse colon; left‐sided, descending colon to the rectum.

Abbreviations: LED, light‐emitting diode; sl., slightly; T1a, slightly invasive submucosal cancer (<1000 μm); T1b, deeply invasive submucosal cancer (1000 μm).

## DISCUSSION

This study showed excellent agreement for the JNET classification comparing images obtained using laser‐BLI and LED‐BLI. A high agreement rate was also shown regardless of endoscopists’ confidence level (high or low), experience, or whether the lesion was neoplastic or not. A high proportion of comparison scores for surface patterns, vessel patterns, and brightness were “almost similar”.

Laser light is a coherent beam with an extremely narrow wavelength range which is an ideal light source for producing NBI. BLI from a laser light source is widely used in clinical practice, mainly in Asian countries. Recently, Fujifilm Corporation developed a breakthrough technology to accurately control the intensity ratio between multiple LEDs. This technology enables the use of multi‐LEDs as a light source in the endoscopy unit. However, the wavelength of narrow‐band light emitted from LEDs is not theoretically as narrow as purely single‐wavelength laser light. Therefore, it should be evaluated to determine if this theoretical difference affects clinical diagnosis using endoscopic narrow‐band light.

The overall rate of agreement for the JNET classification comparing laser‐BLI and LED‐BLI was 92.5% with an excellent weighted kappa value of 0.99. Therefore, LED‐BLI can be used as an alternative to laser‐BLI. The agreement rate for the JNET classification was excellent between laser‐BLI and LED‐BLI regardless of the experience level of the endoscopists, their confidence level (high or low), or whether or not the lesion is a neoplasm, which means that LED‐BLI can be used as an alternative to laser‐BLI regardless of those situations. The JNET classification is determined based on surface and vessel patterns seen at a close view under magnified observation, and therefore any classification is enabled as long as the brightness of the focused area is adequate. However, the paired BLI pictures randomly arranged side‐by‐side in the present study might bias evaluators to assign the same JNET classifications. Since the LECOL study has already shown the non‐inferiority of white light imaging and linked color imaging of LEDs compared to a laser light source,^6^ LED colonoscopy can completely replace laser colonoscopy. Since some countries prohibit “laser” endoscopy, only LED endoscopy can be used in such countries. Therefore, endoscopists can select either.

The distribution of comparison scores shows that almost all endoscopists did not consider either laser‐BLI or LED‐BLI to be superior. The brightness of LED‐BLI was higher than that of laser‐BLI. Non‐experts notably judged “LED better” more often than experts for brightness (8.2% [31/378] vs. 1.1% [4/378]). Our previous report showed that linked‐color imaging using LED light was better for brightness.^6^ The higher score of LED‐BLI for brightness may relate to the high brightness level of the LED colonoscope. Non‐experts in particular may feel that LED‐BLI is more acceptable than laser‐BLI because of its brightness.

There are several limitations to the present study. The number of lesions was not sufficient to reveal statistical non‐inferiority of LED‐BLI to laser‐BLI because this study was originally designed to show non‐inferiority regarding polyp visibility with white light imaging and linked‐color imaging comparing laser and LED light sources. A selection bias for lesions was inevitable because only patients who underwent ESD or EMR were enrolled. In addition, the timing of precheck endoscopy and therapeutic endoscopy was not always on the same day. These two endoscopic examinations were performed within 1 month of each other. The randomly side‐by‐side arrangement of the lesion images might have biased the evaluators to assign the same JNET classifications. The endoscopists evaluated only one still picture of each magnified endoscopic image using laser‐BLI and LED‐BLI, which were different from practical video endoscopic examinations. High‐magnification images were not used in the present study.

In conclusion, the present multicenter study shows excellent agreement for JNET classifications comparing images obtained using laser‐BLI and LED‐BLI. Additionally, the comparison score shows that almost all endoscopist evaluators did not consider either laser‐BLI or LED‐BLI to be superior.

## CONFLICT OF INTEREST STATEMENT

H.S. has received grants from Fujifilm Co. Ltd. H.Y. has consultant relationships with Fujifilm Co. Ltd. and received honoraria, grants, and royalties from the company. N.Y is an editor of Digestive Endoscopy. N.Y. and O.D. received a research grant from Fujifilm Co. Ltd. (J162001222). Y.I. is an executive councilor of the Japanese Society of Gastroenterology. The other authors declare no conflicts of interest.

## Data Availability

Although the patient data supporting the results of this study are available from the corresponding author if requested, some data is restricted by the Institutional Review Board of Jichi Medical University and the Institutional Review Board of the Kyoto Prefectural University of Medicine.
